# Care pathways for people with major depressive disorder

**DOI:** 10.1192/j.eurpsy.2022.1587

**Published:** 2022-09-01

**Authors:** R. Strawbridge, A. Young, Major Depressive Disorder Working Group

**Affiliations:** 1 Institute Of Psychiatry, Psychology & Neuroscience, King’s College London, Centre For Affective Disorders, London, United Kingdom; 2 n/a, N/a, n/a, Belgium

**Keywords:** major depression, care pathways, treatment gaps

## Abstract

**Introduction:**

Major depressive disorder (MDD) is a leading cause of disability worldwide, in part due to its high prevalence and high rates of comorbidities, recurrence, chronicity and treatment-resistance. These indicate that MDD is treated suboptimally despite a multitude of effective interventions and well-regarded best-practice treatment guidelines. To improve the management of MDD, the nature and extent of ‘gaps’ in care pathways need to be understood.

**Objectives:**

We aimed to: 1. Identify ‘treatment gaps’ and patient needs along the care pathway, and determine the extent of these gaps (i.e. discrepancy between best- and current-practice). 2. Propose policy recommendation on how minimise treatment gaps for MDD.

**Methods:**

*Care pathway analysis:* A set of relevant treatment gaps were agreed upon, a priori, based on gold-standard stepped-care guidelines. Data was gathered from a variety of sources in six countries (UK, Sweden, Germany, Italy, Portugal, Hungary). *Policy recommendations:* To attain expert consensus on proposed recommendations, a modified-Delphi approach was undertaken with a multidisciplinary panel of experts across Europe.

**Results:**

Taken together, data indicated that: ˜50% of episodes are undiagnosed, lifetime delay to treatment averages ˜4 years, ˜25-50% of patients are treated at any one time, ˜30-65% are followed up within 3 months of treatment, ˜5-25% can access psychiatric services. 28 specific recommendations to optimise pathways were made to enhance MDD detection (pathway entry), increase multimodal treatment, facilitate continuity of follow-up after treatment and increase access to specialist care.

**Conclusions:**

There are concerning treatment gaps in depression care across Europe, from the proportion of people not being diagnosed to those stagnating in primary care with impairing, persistent illness.

**Disclosure:**

No significant relationships.

